# The state of anxiety treatments for adolescents and adults down syndrome: Results from a scoping rapid review

**DOI:** 10.1016/j.xjmad.2024.100056

**Published:** 2024-02-16

**Authors:** Jill C. Fodstad, Lauren B. Jones, Micah Iticovici, Rachel M. Russell, Molly Bullington, Emily Meudt

**Affiliations:** a Indiana University School of Medicine, USA; b Indiana University Health Physicians, USA; c Cincinnati Children’s Hospital Medical Center, USA

**Keywords:** Down syndrome, Anxiety, Evidence-based treatment, Adults, Adolescents

## Abstract

Adolescents and adults with Down syndrome are noted to display symptoms consistent with various anxiety disorders. While evidenced-based practices, including psychotherapies and psychopharmacology, exist and effectively treat anxiety in neurotypical populations, less is known about anxiety treatments for persons with Down syndrome. A scoping rapid review was conducted in April 2023 to determine what treatments are being used to target anxiety in adolescents and adults with Down syndrome, the quality of those treatments, and their alignment with current evidence-based practices. A total of eleven articles, primarily single case or case series, published between 1981 and 2022 were identified targeting adolescents and adults with Down syndrome diagnosed with specific phobias, selective mutism, generalized anxiety disorder, agoraphobia with panic, and non-specific anxiety symptoms. Interventions used most often aligned with evidence-based anxiety treatment guidelines and included psychotherapy, complementary and alternative medicine, and psychopharmacology. While most studies reported positive treatment responses showing reductions in anxiety symptoms post-treatment, the quality and generalizability of the studies was primarily poor. More rigorous research evaluating the effects of treatment for anxiety symptoms in the DS population are needed to develop guidelines to address anxiety disorders in this vulnerable population.

## 1 Introduction

Down syndrome (DS) affects between 1 in 1000 and 1 in 1100 live births worldwide [1], [2] and is the most common genetically linked cause of intellectual disability. It is estimated that between 15–40% of adults with DS have a co-morbid neurobehavioral or psychiatric condition [3]. Co-occurring anxiety disorders and anxiety-related behaviors are noted to frequently occur in DS adolescents and adults [4].

Data suggests that while anxiety disorders may be equal to or less common in individuals with DS compared to individuals with other neurodevelopmental disabilities (e.g., intellectual disability without DS, Williams syndrome; [5], [6]), anxiety disorders in the DS population are still common and may be equally as prevalent or more prevalent than in the general population [4], [7]. For individuals with DS, having a moderate or major mental health condition predicts a lower quality of life [8]. Additionally, anxiety disorders have been specifically shown to negatively impact the performance, skills, and ability to complete daily living skills for individuals with intellectual disabilities, including DS [9]. Thus, it is imperative to identify and treat anxiety disorders in the DS population.

Currently, evidence-based treatments for anxiety exist for neurotypical populations, including both psychotherapeutic and pharmacological interventions. Regarding psychotherapeutic interventions, cognitive behavioral therapy (CBT) is the treatment approach with the most robust evidence-base and is the gold-standard treatment for anxiety [10]. However, third-wave therapies including mindfulness-based training [11], acceptance and commitment therapy [12], and dialectical behavioral therapy [13] have also been shown to have promising effects on treating anxiety disorders in the general population. For individuals with DS and co-occurring anxiety disorders, it is unclear what psychotherapeutic strategies are being used and if the strategies that are currently being used align with what has been shown to be effective in neurotypical individuals.

Regarding pharmacotherapy, selective serotonin reuptake inhibitors (SSRIs) and selective serotonin norepinephrine reuptake inhibitors (SNRIs) are recommended as first-line anxiety treatments for the general population [14]. Additionally, some benzodiazepines have shown positive results in reducing anxiety symptoms [15], though these medications are not recommended for routine care [14]. Other pharmacological approaches to anxiety management for the general population include buspirone, moclobemide, pregabalin, and tricyclic antidepressants. Similar to our lack of knowledge about psychotherapeutic strategies used to treat anxiety in the DS population, it is unclear what pharmacological treatments are being used and if the approaches being used align with the broader evidence-based treatments.

To aid in our understanding of evidence-based guidelines for treating anxiety in individuals with DS, information is needed to understand the current evidence base and identify necessary future directions for psychotherapeutic and pharmacological interventions for co-morbid anxiety disorders in DS. Thus, the goal of this study was to identify via a rapid scoping review what strategies have been employed to clinically treat anxiety and anxiety-related disorders or behaviors in adolescents and adults with DS. Specifically, the study aims were to 1) identify strategies used to target and treat symptoms of anxiety or anxiety-related behaviors, 2) determine the alignment of these strategies with current standards for evidence-based anxiety treatment; 3) assess the quality of the available research, and 4) identify the presence and type of any knowledge gaps. A final aim of this project was to aid in stimulating more research to further develop evidence-based treatments for anxiety and anxiety-related disorders in adolescents and adults with DS.

## 2 Method

In April 2023, a scoping rapid review was conducted to identify anxiety treatments targeted for DS adolescents and adults. The following inclusion criteria were used: 1) participants must be aged 13 years or older, 2) participants must have a diagnosis of DS (if the study included a case series or group study at least one participant must have DS), 3) participants must have co-occurring anxiety (e.g., excessive worry, Generalized Anxiety Disorder, Specific Phobia, Social Anxiety Disorder, Agoraphobia, Anxiety Disorder), 4) treatment must target anxiety symptoms (not OCD/”obsessional or compulsive” behaviors), and 5) study must be written in English. Non-published theses or dissertations were eligible for inclusion, but literature reviews, editorials, posters, book chapters, and opinion pieces without a clear case study were excluded. Additionally, studies were excluded if they did not meet the inclusion criteria.

### 2.1 Literature search and article selection

Using the Preferred Reporting Items for Systematic Reviews and Meta-analyses (PRISMA) standards [16], the following databases were searched including PubMed, CINAHL, EMBASEE, PsycINFO, and Scopus (Medline). The search terms included combined terms related to anxiety (e.g., “anxiety disorders,” “generalized anxiety,” “social anxiety”), down syndrome (e.g., “down syndrome,” “trisomy 21”), and treatment (e.g., “therapy”, “treatment”, “intervention”). When searching PubMed, MESH terms were used for “down syndrome,” “anxiety disorder,” and “behavioral disciplines and activities.” Search limits were applied for age (i.e., 13 years or older), subjects (i.e., human subjects only), and language (i.e., English only). After conducting the initial search in each database, titles and abstracts were screened to determine if the study met inclusion criteria. The remaining full-text articles were then read and any study that did not meet the inclusion criteria was eliminated. All titles, abstracts, and full-text articles were read and evaluated by two independent raters (IRR = 100.0%).

### 2.2 Data extraction and coding

Once it was determined which full-text articles met inclusion criteria, two raters extracted data on an a-priori defined spreadsheet in which background information and data to support key aims were coded. The following background information was coded: first two authors, publication year, number of participants in the study with DS, age of participants, psychiatric diagnoses, medical comorbidities, source of subjects (e.g., hospital), methods (e.g., observational, response to treatment), and study design (e.g., case study, experimental study). Additionally, the following data to support key aims was coded: type of intervention, length of intervention, medication dose/optimization, adverse reactions to medications, primary outcome measure, informant who completed outcome measure, symptom change, maintained gains at follow-up, and limitations of each article. A quality assessment was also completed using an adapted Cochrane Risk of Bias tool for intervention studies (for further information see single-case design risk of bias (SCD RoB) tool [17]). The SCD RoB tool serves as a guide to assess each study for sources of potential bias across eight subdomains within the broader categories of selection bias, performance bias, and detection bias. Using the SCD RoB tool, each study was rated for its risk of bias (i.e., low, unclear, or high) across each of the eight subdomains (i.e., sequence generation, participant selection, blinding of participants/personnel, procedural fidelity, blinding of outcomes assessment, selective outcome reporting, dependent variable reliability, and data sampling) by the two independent raters (IRR = 92.3%).

## 3 Results

. summarizes studies that were retained at each step of the rapid review. Additionally, a summary of relevant study information can be found in Table 1. In total, eleven studies met inclusion criteria and studies ranged in publication year from 1981 to 2022. Studies included a substantial proportion of case reports (n = 7), two randomized control trials, one case series, and one non-randomized control trial.

**Fig. 1:**
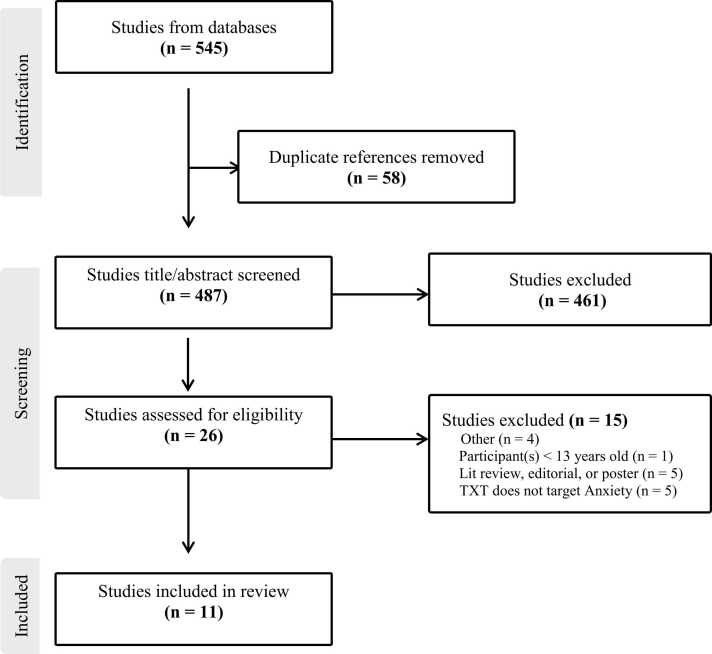
Search Strategy and Results.

**Fig. 2:**
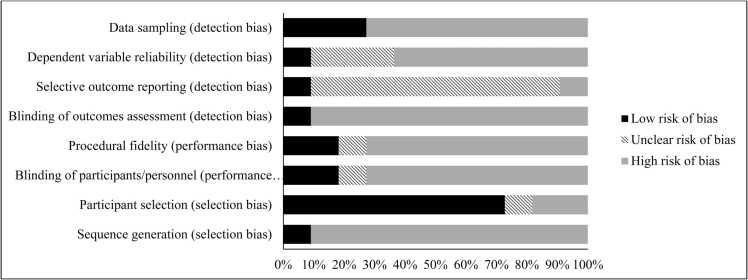
Risk of Bias Across Articles.

**Table 1:** Study Characteristics.

	N with DS. Age range (yrs.)	Diagnosis	Study Type	Txt Type	Txt Details	Anxiety Measure Used	Txt Outcome on Anxiety Symptoms
Bell et al., 2003	1; 36 yrs.	Selective mutism	Case report	Trp	Reinforcement and graduated speech prompting (22 sessions)	Observed sound production and participation in group (clinician rated)	Improvements in quality of life at home and community-peer social interactions reported. increases in time spent in group and number of whispered words.
Cooney et al., 2017	5 in active txt; ages 23-60 yrs.	Recurrent anxiety and depression	RCT	Trp	Computerized CBT (cCBT) game or 7-week treatment as usual (TAU)	Glasgow Anxiety Scale for people with an Intellectual Disability (GAS-ID; self-report)	cCBT arm with increased group x time interaction vs. TAU with respect to changes in GAS-ID scores.
Cowdrey & Walz, 2014	1; 39 yrs.	Specific phobia	Case report	Trp	11-sessions of Modified CBT with graduated exposures, augmented with mindfulness and bereavement work	Modified Abbreviated Spider Phobia Questionnaire (SPQ-15; self-report)	SPQ-15 decreased from 10 to 2 (out of 15 total point) post-txt with increased ability to handle small spiders reported.
Freeman et al., 1997	1; 31 yrs.	Specific phobia	Case report	Trp	Behavioral relaxation training, modeling, and graded exposure (40-step procedure)	Staff-report scale of observed anxiety and community activity attendance	Pre-txt exposure anxiety on 28 of 40 steps decreased to 6 of 40 steps post-txt with co-occurring 86% increase in community engagement.
Howe et al., 2022	3; 16-29 yrs.	GAD (n = 3); social anxiety (n = 1)	Case series	Pharm	Buspirone optimized up to 10 mg (n = 1), 20 mg (n = 1), and 22.5 mg (n = 1)	Clinician observation and Pediatric Anxiety Rating Scale (PARS; retrospective parent-report)	*Case 1:* decreased anxiety and improved function and adaptability; gains maintained at 2-year follow-up. PARS decreased from 33 to 10.; *Case 2:* decreased anxiety and impairment reported; gains maintained at 2.5-year follow-up; PARS decreased from 28 to 12.; *Case 3:* decreased anxiety and increased functioning; gains maintained at 2.5-year follow-up; PARS decreased from 30 to 10
Hurley et al., 2004	1; 34 yrs.	Agoraphobia with panic; multiple specific phobias	Case report	Trp + Pharm	Modified CBT (27 sessions); 20 mg of fluoxetine(discontinued due to side effects), 20 mg of paroxetine (discontinued due to ineffectiveness), 1 mg of lorazepam given for a flight	Qualitative observations on behavior change (reporter not specified)	Patient had less avoidance and increased independence but maintained specific phobias
Kneebone & Al-Daftary,2006	1; 32 yrs.	Specific phobia	Case report	Trp	In-vivo flooding exposure (single session lasting 105 min)	Time spent in physiotherapy txt without distress	Increased tolerance from 10 s to 45 min of physiotherapy txt, maintained for 68 sessions. No distress noted at 5-9 months of follow-up
Kollar et al., 2018	30; age not reported	Anxiety during dental visits	Non-randomized control trial	CAM	Single 10-minute photoacoustic sessions with either rhythmic sounds or relaxation music	Speilberger STAI child version (STAI-S-C and STAI-T-C); Dental Anxiety Scale (DAS); Dental Anxiety Question (all self-report)	Rhythmic sounds showed a statistically significant reduction in anxiety on all measures. Relaxation music showed a non-statistically significant decrease in anxiety on STAI-C.
Millar & Greenhill, 2022	1; late 30 s	No diagnosis, but anxiety symptoms present	Case report	Trp	Adapted CBT (virtual)	Glasgow Anxiety Scale for people with an Intellectual Disability (GAS-ID; self-report)	GAS-ID decreased from 24 to 16 at mid-point and endpoint respectively with self and parent reported improved mood and confidence.
Perić et al., 2022	25; 15-17 yrs	Anxiety and/or depression	RCT	CAM	Adapted soccer program (32 sessions)	Study-designed anxiety and depression scale rated by educator and parent	Significant group X time interaction was found favoring the exercise group over the control group. Exercise group showed significant improvement in anxiety and depression symptoms
Waranch et al., 1981	1; 21 yrs.	Specific phobia	Case report	Trp	In vivo desensitization and shaping (20 weeks with 5-week assessment period)	Clinician-report # of active and passive approaches to phobic stimulus; Parent-report # compliance with real-world exposures	100% approach response to various phobic stimuli, improved compliance with real-world exposures maintained at 6 months.

*Note:* GAD = generalized anxiety disorder; Txt = treatment; CBT = cognitive behavior therapy; RCT = Randomized Control Trial; CAM = complementary and alternative medicine; Pharm = pharmacotherapy; Trp = psychotherapy

### 3.1 Anxiety treatments used and alignment with evidence-based strategies

Most of the studies in this review reported on psychotherapeutic interventions for anxiety in the DS population (n = 7). Additionally, one study evaluated a pharmaceutical intervention [18], one study reported on both pharmaceutical and psychotherapeutic interventions [19], and two studies tested complementary and alternative medicine approaches [20], [21].

Of the eight studies that reported psychotherapeutic interventions, four of the studies used modified or adapted CBT [19], [22], [23], [24], which often included many of the common elements of CBT such as psychoeducation [23], exposures [23], mindfulness/relaxation exercises [19], [22], [23], coping strategies [24], coping statements [19], and homework assignments [19]. In addition, modifications were included to fit the needs of individuals with DS such as the use of visuals and/or social stories [22], [24], frequent repetition [24], modification of language used [24], inclusion of caregivers or staff in treatment [19], [24], and assessment of suitability for CBT principles prior to starting treatment [23], [25].

Generally, the approaches for treating anxiety across these studies were aligned with the current standards for evidence-based anxiety treatment, as CBT is seen as the current gold-standard treatment for anxiety [10]. Further, making modifications to CBT when working with individuals with developmental disabilities is well-supported in the literature and has been shown to lead to reductions in anxiety for individuals with autism spectrum disorder [26] and intellectual disability [27].

Four additional studies relied heavily on behavioral strategies and less on the cognitive elements of modified CBT [28], [29], [30], [31], which is a common modification made when working with individuals with developmental disabilities [26]. Three of these studies presented treatments for specific phobias and had an emphasis on exposure-based therapy including behavioral relaxation training, modeling, and graded exposure [29], in vivo desensitization and shaping [31], and a single-session in-vivo flooding exposure [30]. The other study that emphasized behavioral techniques used reinforcement and graduated speech prompting to encourage speech and sound production in an individual with selective mutism [28].

Similarly, many of these studies used approaches for treating anxiety that are aligned with the current standards for evidence-based anxiety treatment. In line with the techniques used in Freeman et al. [29], Kneeborne and Al-Daftary [30], and Waranch et al. [31], a review by Choy et al. [32] found that in vivo exposures were the most efficacious treatment for specific phobias. Additionally, meta-analyses have shown that exposure therapy leads to treatment effects that are more robust than other psychotherapies and placebo [33]. Regarding the single-session, flooding exposure used by Kneeborne and Al-Dattary [30], meta-analytic results have found no difference in the effectiveness of single-versus multi-session exposure [34], however, less is known about the differences in effectiveness between flooding and graduated exposure within a single session.

The literature on treatment for selective mutism is less robust, however, a review of treatments found that the most common approaches used were behavioral and systems based which included strategies such as shaping, hierarchical exposure, contingency management, stimulus fading, adult skills training, psychoeducation, and consultation [35]. The reinforcement and graduated speech prompting techniques used in Bell et al. [28] would fall in line with some of these approaches but may not be as comprehensive as other treatments for selective mutism (i.e., no inclusion of exposure or adult skills training).

The remainder of the studies included in the review reported on either pharmacotherapy (n = 2) or complementary and alternative medicine-based approaches (n = 2). Specifically, Howe et al. [18] reported on the effects of buspirone and was the only study to evaluate pharmacotherapy only. In the literature, buspirone is typically recommended as a second-line treatment for anxiety after SSRIs and SNRIs [36]. However, the efficacy of buspirone has been demonstrated in patients with GAD [37] and has been recommended for the treatment of anxiety in individuals with DS [38]. Thus, the approach used in Howe et al. [18] may be less in line with the broader evidence-base but may be beneficial for individuals with DS. Hurley et al. [19] reported on the use of pharmacotherapy in addition to psychotherapy and used the traditional first-line treatments for anxiety that are recommended for neurotypical individuals in the literature (i.e., SSRIs). Lorazepam, a benzodiazepine, was also given for a singular flight. The literature shows that benzodiazepines are not recommended for routine use [15] and lorazepam is best used on a short-term and as-needed basis [39].

Finally, Perić et al. [21] and Koller et al. [20] reported on complementary and alternative medicine-based approaches. Although psychotherapy and pharmacotherapy are typically considered to be first-line treatments for anxiety, the broader literature includes empirical support for both approaches. In line with Perić et al.’s [21] adapted soccer program, there is evidence to support that physical activity can lead to reductions in anxiety for both the general population [40] and for individuals with intellectual disability [41]. In line with Koller et al.’s [20] test of photoacoustic stimulation before a dental procedure, significant differences in dental anxiety have been found based on audiovisual distraction in individuals with [42] and without [43] intellectual disability.

### 3.2 Quality appraisal of included studies

To confidently interpret results, the quality and rigor of research studies must be thoroughly considered. Specific issues noted in the articles include lack of follow-up data, single-case design, use of non-validated measures, and use of subjective measures/outcomes.

Regarding follow-up data, it is imperative that studies include post-treatment outcome data to help establish the long-term efficacy of the treatment. There is no official recommendation as to what length of time is sufficient to observe any changes in patient response, nor is there official guidance with regards to what a reasonable follow-up period might be. However, many treatments take 4–6 weeks to elicit a response and it is recommended that clinicians continue to follow patients at least 12 months after initial treatment [44]. Of the eleven articles, six did not include any follow-up data. Lack of follow-up has considerable impact on a study’s validity and limits the ability to make conclusions surrounding the long-term efficacy and safety of treatments provided [45], [46]. In addition, it also makes it difficult to assess if termination of the treatment has any effect on the patient’s symptoms.

Another significant consideration is the study design. Of the eleven articles, eight were case reports or case series. While these study designs play an important role in identifying symptoms, making a diagnosis, and detailing treatment options for uncommon or atypical cases, they are at risk of significant bias, as they lack the reliability and external validity necessary to make accurate assessments and predictions of future treatment response in other individuals of a similar population [47]. Additionally, there is an added difficulty in the DS population due to the massive amount of diversity in presentation and unique comorbidities. As a result, many of the case reports and case series report on specific problems in patients with unique circumstances and may be less applicable to the broader DS population.

Finally, it is important to assess the method quality of the included studies to ensure that the methods used are appropriate for reliably measuring the outcome in question. Unfortunately, across the eleven articles evaluated, there were several methodological flaws identified, reflecting overall poor research quality. Many of the studies included measures which were not validated to assess for treatment response, including using novel clinician-designed scales or tasks, that rely on clinician evaluations, or using retroactive reports of symptoms. More specifically, five of the studies did not employ any objective or validated measurement tool.

Using non-validated measures significantly impacts the validity of a study as there is no data to suggest that the measure is identifying and assessing symptoms appropriately, which can result in inaccurate conclusions regarding the efficacy of treatments and expected treatment-response [48], [49]. Similarly, due to known discrepancy between memory and experience, the use of retroactive report and qualitative measurement introduces various biases which impact the ability to accurately interpret treatment responses [50]. In addition, with longer periods between retrospective reports, the data is subject to recency biases which could significantly affect outcomes.

## 4 Discussion

The goal of the current study was to identify via a rapid scoping review the strategies that have been employed to treat anxiety and anxiety-related disorders in adolescents and adults with DS. Overall, the current study identified a total of 487 articles discussing anxiety treatment in patients with DS. Of these, eleven studies met inclusion criteria and were further analyzed. Results revealed that across the studies reviewed, treatments used for patients with DS most often included strategies aligning with current standards for evidenced-based anxiety treatment, including (modified) CBT, behavioral strategies, and pharmacotherapy. While many of the studies reported positive treatment responses showing reductions in anxiety symptoms post-treatment, the available research was sparse, and the quality and generalizability of the studies was primarily poor.

Specifically, it is notable that only eleven studies met inclusion criteria, despite there being no restriction on publication year. This indicates that the available research for the treatment of anxiety in individuals with DS is currently minimal. Further, most of the studies were case reports or case series. Although case reports and case series can serve as an excellent way to advance our knowledge in the field, they are uncontrolled study designs that have a much greater risk of bias compared to well-controlled studies [51]. Without rigorous randomized control trials, it is difficult to draw generalizable conclusions on the efficacy of these treatments for individuals with comorbid anxiety and DS.

It is also important to note that only four out of eleven studies included a self-report measure of anxiety for individuals with DS. Although parent, clinician, and staff report can be beneficial, it is imperative that future research clarify the perspectives of individuals with DS on changes in their own anxiety. Additionally, there were many methodological flaws with the measures being used including measures not being validated, symptoms only being reported retroactively, and qualitative observations being the only measure of change. It is imperative that future studies include well-validated measures including rigorous quantitative and qualitative analyses so that the effects of these treatments can truly be understood. Finally, only five out of eleven studies reported follow-up data. Thus, it is still largely unknown whether the effects of these treatments persist over time.

### 4.1 Limitations and future directions

There are notable limitations of the current rapid review that are important to consider when interpreting these results. First, the results in each study were reported on qualitatively. Thus, no rigorous meta-analytic strategies were used to analyze the treatment effects across the included studies. Although qualitatively assessing the state of the literature is an important first step, more rigorous meta-analytic strategies would be helpful in further assessing and understanding the magnitude of treatment effects. Similarly, beyond reporting the outcomes provided on patients’ symptoms and the treatment methodology used, the current study did not thoroughly analyze the extent to which these studies were following evidence-based guidelines for treatment. Further, many of the treatments used employed modifications or additional components to increase the likelihood of successful outcomes for the DS individual such as visuals and/or social stories, frequent repetition, tangible reinforcers and/or verbal praise, modeling, simplifying language, and including caregivers in treatment. While these additional components did appear to aid in positive outcomes, it is unclear whether these treatments with modifications could be considered evidence-based care for the DS population pending further investigation, or if new treatments are needed. Our rapid review indicated that there is not enough research in this area to be able to make that determination currently. Until further research occurs, clinicians should continue to follow established anxiety treatment guidelines for the neurotypical population when individuals with DS present with anxiety and anxiety-related behavior. However, going forward, it will be imperative for future research to assess the effects of anxiety treatments in the DS population using more rigorous methodology, so that conclusions about treatment effectiveness and recommendations for evidence-based care can be made. Subsequently, it will be imperative to develop guidelines for providers on what can be considered evidence-based care for anxiety in this population.

Finally, the current rapid review only reviewed studies in which the patients were 13 years and older. It is imperative that future research review the current strategies that have been employed to treat anxiety and anxiety-related disorders in children with DS, especially when thinking about prevention efforts for later development of anxiety disorders in adolescents and adults.

## 5 Conclusion

Outcomes of the current rapid review on treatments for anxiety and anxiety-related behaviors in adolescents and adults with DS found that interventions used most often aligned with current standards in neurotypical populations for evidence-based anxiety treatment, but with noted modifications. However, the poor quality and rigor of the included studies and the overall lack of research in this area limits the ability to interpret the true efficacy of these treatments. In the future, rigorous research designs evaluating the effects of psychopharmacologic and behavioral treatment for anxiety symptoms in the DS population are needed to develop evidence-based guidelines to address anxiety disorders in persons with DS.

## CRediT authorship contribution statement

The authors have no disclosures and no competing interests. This research did not receive any specific grant from funding agencies in the public, commercial, or not-for-profit sectors. This paper, or a portion of this paper, is not presently under consideration for publication nor has been published in any other peer reviewed or non-peer reviewed publication.

## Declaration of Competing Interest

The authors declare that they have no known competing financial interests or personal relationships that could have appeared to influence the work reported in this paper.
